# CORRIGENDUM

**DOI:** 10.1111/1759-7714.14467

**Published:** 2022-06-02

In Sun *et a*l.^1^ the following error was published on page 1594.

The authors found out that figure 2b and figure 4b were mixed and repeated. The updated figure 2b is shown below:

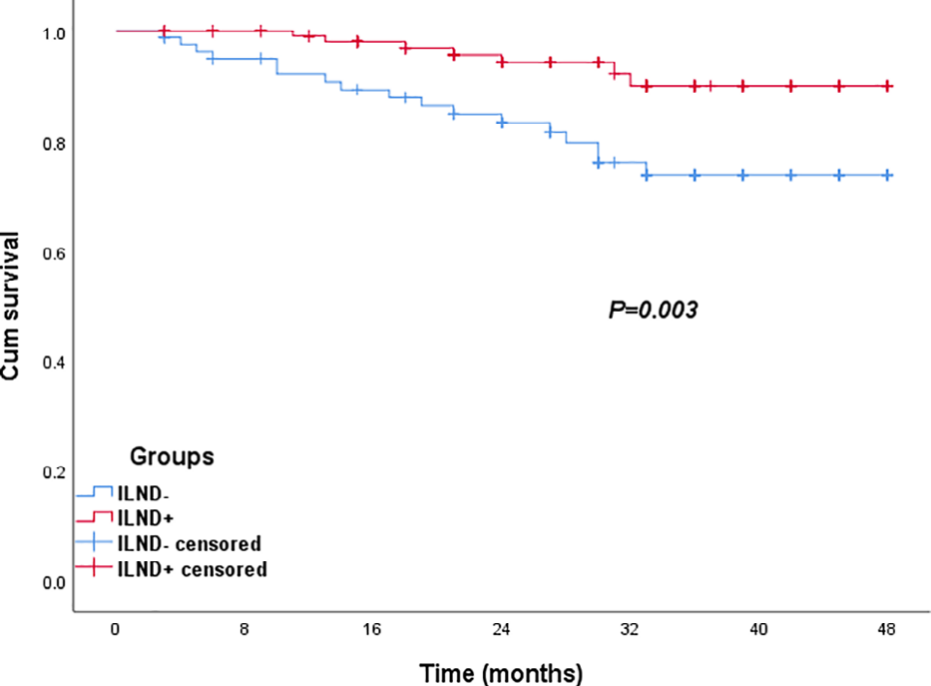

The authors apologize for the error and any inconvenience it may have caused.
